# Are Limits of Concern a useful concept to improve the environmental risk assessment of GM plants?

**DOI:** 10.1186/s12302-017-0104-2

**Published:** 2017-02-16

**Authors:** Marion Dolezel, Marianne Miklau, Andreas Heissenberger, Wolfram Reichenbecher

**Affiliations:** 1 Environment Agency Austria, Spittelauer Laende 5, 1090 Vienna, Austria; 2 Federal Agency for Nature Conservation, Konstantinstrasse 110, 53179 Bonn, Germany

**Keywords:** GMO, Genetically modified plants, Environmental risk assessment, Environmental harm, Damage thresholds, Limits of Concern

## Abstract

**Background:**

The European Food Safety Authority (EFSA) has introduced a concept for the environmental risk assessment of genetically modified (GM) plants which foresees the definition of ecological threshold values defining acceptable adverse effects of the GM plant on the environment (Limits of Concern, LoC).

**Methods:**

We analysed the LoC concept by scrutinising its feasibility with regard to important aspects of the environmental risk assessment. We then considered its relationship with protection goals, the comparative safety assessment and the stepwise testing approach. We finally discussed its usefulness for assessing long-term effects, effects on non-target organisms and species of conservation concern.

**Results:**

The LoC concept is a possible approach to introduce ecological thresholds into environmental risk assessment in order to evaluate environmental harm. However, the concept leaves many important aspects open. Thresholds for environmental harm for protection goals need spatial and temporal differentiation from LoCs used for ERA indicators. Regionalisation of LoCs must be provided for as biodiversity levels and protection goals vary across the EU. Further guidance is needed with respect to the consequences, in case LoCs are exceeded and a link needs to be established between environmentally relevant results from the comparative safety assessment and the LoC concept. LoCs for long-term effects have to be evaluated by long-term monitoring. LoCs for non-target organisms need to be discriminated according to the species and parameters assessed.

**Conclusions:**

The overall LoC concept is considered useful if LoCs are further specified and differentiated. Although LoCs will finally be determined by political decisions, they should be based on scientific grounds in order to increase confidence in the conclusions on the safety of GM plants.

## Background

In the European Union, genetically modified organisms (GMOs) need to undergo an authorisation procedure in which an environmental risk assessment (ERA) is performed in order to conclude on potential risks to the environment. The European Food Safety Authority (EFSA) and its GMO Panel play a crucial role in this authorisation procedure as they issue, based on data provided in the GMO applications, scientific opinions on the safety of GMOs and provide advice for risk managers (i.e. the European Commission and the EU Member States). In addition, the GMO Panel produces guidance documents to specify certain aspects of GMO risk assessment and to provide guidance for the preparation and presentation of GMO applications.

For the risk assessment of GMOs and derived food and feed, EFSA suggests the use of the so-called ‘comparative safety assessment’ as a starting point for the whole risk assessment process [1, 2]. By this concept, a comparator, usually a non-GM plant with a similar genetic background is used for comparison when assessing intended and possibly unintended effects of the GMO. This assessment comprises compositional parameters and other plant characteristics such as agronomic or phenotypic parameters. For the ERA, the comparative safety assessment should also use information on plant–environment interactions of the GMO [3]. The concept behind the comparative safety assessment is the assumption that conventionally cultivated plants are safe for consumers, animals and the environment. Their ‘history of safe use’ should therefore assist the safety evaluation of a novel or GM food [2, 4, 5].

As a hitherto practice in the risk assessment, any statistically significant differences found in the comparative assessments, which were not intended by the genetic modification, were classified by the applicants as being ‘biologically not relevant’. This is due to the fact that the observed values were often lying within the range of values reported for conventional non-GM plant varieties in the scientific literature. According to the claimed history of safe use the GMOs were considered safe with no further evaluation of their potential consequences for risks to human health or the environment [6].

In 2010, EFSA published a guidance document for the ERA of GMOs [3]. The aim of this document was to further develop and update the guidelines of the ERA previously available [1]. In the guidance document, EFSA requires that the biological relevance of statistically significant differences between the GMO and the non-GM comparator should be assessed, also considering potentially hazardous environmental implications [3]. EFSA clearly recognises that such differences could be linked to morphological alterations or metabolic perturbations or may indicate unintended effects which may lead to environmental harm. Any identified potential adverse effects should be linked to assessment endpoints in order to quantitatively evaluate the potential environmental harm. These assessment endpoints are then translated into measurement endpoints for which a Limit of Concern (LoC) has to be defined. EFSA defines Limits of Concern as ‘*the minimum ecological effects that are deemed biologically relevant and that are deemed of sufficient magnitude to cause harm*’ [3]. By testing whether the observed effect falls within the LoC, the biological relevance of the observed effect is determined. According to EFSA, LoCs can be derived from literature data, baseline data, modelling, existing knowledge, or policy goals and shall be explicitly stated and justified by the applicant [3]. The setting of the LoC and the definition of environmental harm are therefore considered crucial for the ERA of a GMO and in particular also for the assessment of potential effects on non-target organisms [7]. The necessity to set thresholds when determining acceptability of risks has also been recognised in international guidelines for the risk assessment of living modified organisms [8].

In this article, a range of questions regarding the operationalisation of the LoC concept for the ERA of GMOs is addressed which arose during the course of the project and, in particular, from expert workshops and interviews (see “Methods” section).

## Results and discussion

### Limits of Concern and protection goals

#### The EFSA guidance

In the first step of the ERA, the applicant has to identify those environmental aspects that need protection from harm and could be negatively affected by the GMO in question [3]. EU-wide relevant legislation defining relevant environmental protection goals are listed comprising biodiversity as well as ecological functions [3]. A direct link between the relevant protection goal and the LoC has to be established by the definition of the so-called assessment endpoints for the relevant protection goal, e.g. a specific biological function or non-target organism group, for which specific measurement endpoints are defined (e.g. mortality). For these measurement endpoints, LoCs should be defined [3].

#### Analysis of the guidance and suggestions for improvements

According to EFSA, protection goals are defined as natural resources or natural resource services whose protection is laid down in legislative acts at EU level [3]. One prominent example is the protection of biodiversity which is agreed by a range of European Directives, Regulations, Action Plans, strategies and conventions [3].

One of the major challenges with respect to the ERA of GMOs is the lack of definition of the desired status and/or condition of the natural resources or natural resource services listed in the legislative acts. Exemptions are the Fauna–Flora-Habitat Directive (Council Directive 92/43/EEC) and the Birds Directive (Directive 2009/147/EC) for which the objective of maintaining or restoring natural habitats and species of Community interest at a favourable conservation status is defined by Community law. In addition, the legislative acts do not contain any specifications of environmental harm for the protected natural resources or resource services. Directive 2004/35/EC refers to environmental harm as effects that are characterised by being adverse, measurable and significant to the relevant protection goals [7]. As a consequence, the delineation of an adverse effect and environmental harm requires the setting of a threshold delimiting negligible from significant adverse effects, commonly termed ‘damage thresholds’ [9].

In practice, the definition of environmental harm and where to set the threshold for significant adverse effects is not based on scientific grounds only. For example, no fixed thresholds are available for the assessment of significant adverse effects on natural habitats protected under Council Directive 92/43/EEC (i.e. Natura 2000 areas). The decision on whether or not a plan or project may compromise the area’s protection objectives is taken case-by-case and is subject to expert judgement [10]. Consequently, also the minimum level of biodiversity to be preserved in agro-ecosystems is a policy objective and may vary considerably between Member States [7].

Clear policy objectives are certainly missing when it comes to define which environmental risks are acceptable and what constitutes environmental harm for GMO risk assessment [11, 12]. Even though EFSA has prioritised protection goals by listing relevant EU environmental and agricultural legislative acts in their guidance documents [3, 7], the policy objectives underlying these acts are still to be made operational, e.g. by suggesting acceptable thresholds for damages caused by GMOs. It has to be emphasised that damage thresholds for EU-wide protection goals should ideally cover multiple environmental stressors [13]. This is also reflected by attempts to operationalise protection goals for risk assessment purposes for several different environmental stressors by the use of the ecosystem services concept [13–15].

It is important to acknowledge the difference between the environmental harm to EU-wide protection goals and the threshold level for acceptable adverse effects used in ERA testing. It is not feasible to assess *ex*-*ante*, i.e. during the pre-market ERA, the potential harm for a relevant EU-wide protection goal due to GMO cultivation. Therefore, indicators are needed to test for potential effects on the relevant protection goals in the ERA [16]. For these indicators, thresholds of acceptable adverse effects can then be defined [17]. In this context, it has to be taken into account that the type and magnitude of potential environmental effects depend on the scale of GMP adoption which cannot be reflected by small-scale and short-term risk assessment studies [18]. Hence, ERA methodologies have to be applied such as modelling approaches which integrate the accumulation of adverse effects across larger spatial and temporal scale resulting in predictions of risks when large-scale and long-term cultivation (over the full authorisation period of 10 years) of GMPs is envisaged [see e.g. 19].

**Fig. 1:**
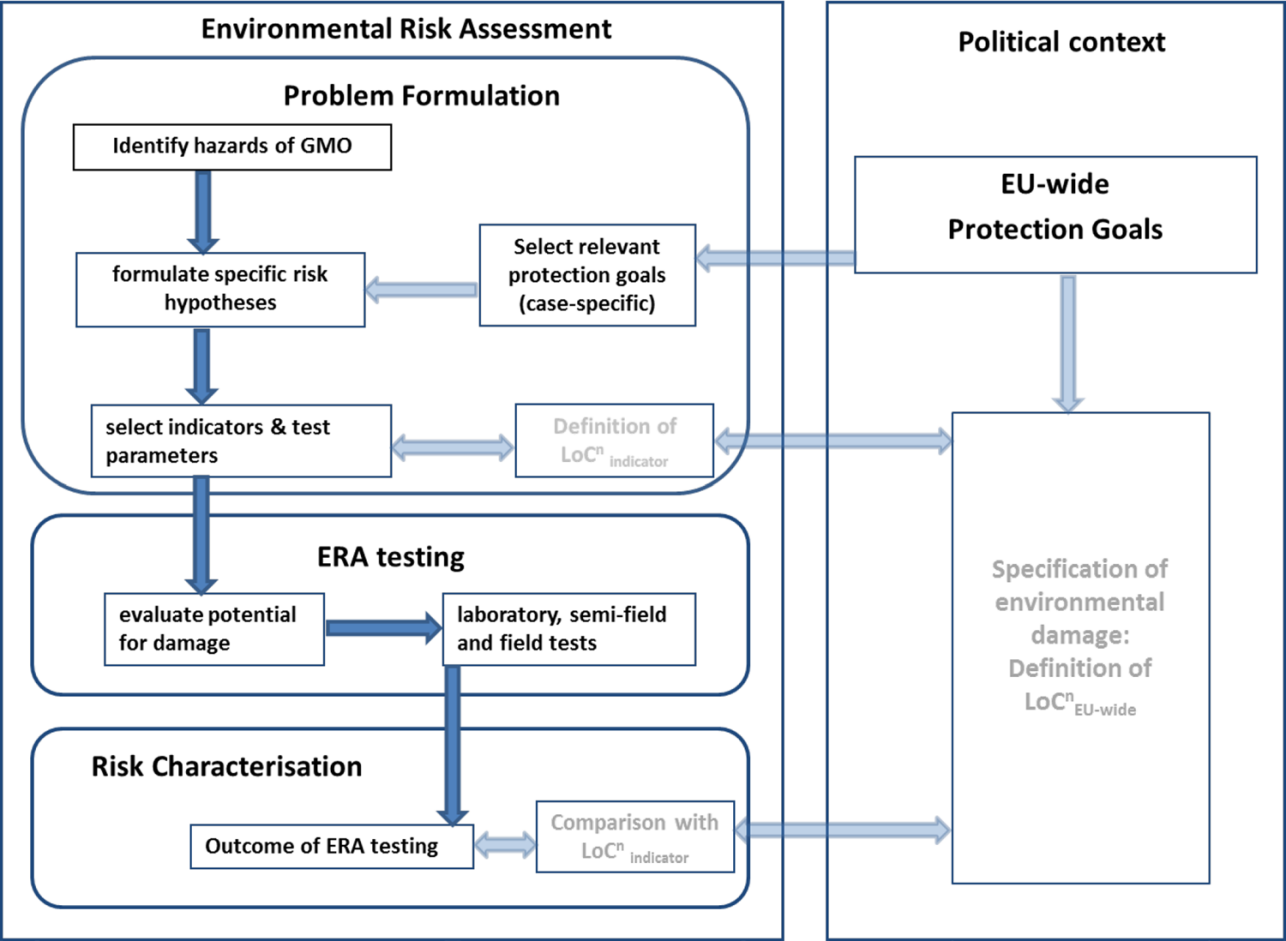
Suggestions for the use of the LoC concept in the ERA of GMOs. *Boxes* and *dark blue arrows* indicate aspects and flow of information as currently done in the ERA. *Transparent boxes* and *arrows* indicate aspects and information flow that still need to be defined. ${\text{LoC}}_{{_{\text{EU-wide}} }}^{\text{n}}$—set of LoC for a natural resource or resource service at EU level. ${\text{LoC}}_{\text{indicator}}^{\text{n}}$—set of LoC for indicators used for ERA purposes

Ideally, and as a first step to operationalise the LoC concept, normative specifications for environmental damage and damage thresholds would have to be defined. For each protected natural resource or resource service at EU level, one LoC or several LoCs (LoC^n^) should be defined (${\text{LoC}}_{\text{EU-wide}}^{\text{n}}$; see Fig. 1). LoCs different from those at EU level have to be specified for testing effects during the ERA by the use of indicators [17]. These LoCs are termed LoC_indicator_ and are used at laboratory, semi-field or field level (Fig. 1). It is probably challenging to link the thresholds for environmental damage at EU level (the LoC_EU-wide_) with those at the ERA testing level (LoC_indicator_). This is notably due to the differences in the spatial and temporal scales for the maximum tolerable adverse effects between the protection goal level and the different ERA testing levels (lab, semi-field and field). If definitions for environmental damage or maximum tolerable adverse effects refer to EU-wide protected natural resources (e.g. the EU-wide population of a certain taxon), then the spatial and temporal scales refers to the whole EU population. In contrast, thresholds for the acceptability of adverse effects at the ERA level need to relate to the meta-population of the same taxon affected when conducting laboratory tests or during field testing.

Figure 1 shows the necessary integration of the LoCs in the current ERA system of GMOs. Once the damage thresholds for protection goals at EU level (${\text{LoC}}_{\text{EU-wide}}^{\text{n}}$) have been defined, damage thresholds for the relevant indicators for ERA testing (the ${\text{LoC}}_{\text{indicator}}^{\text{n}}$) can be set. Once the results of the ERA testing are available, the outcomes for each indicator are compared with the LoC_indicator_ for each testing level.

### Limits of Concern and receiving environments

#### The EFSA guidance

The consideration of the receiving environments where the GMO will be grown and where transgenes may spread is one of the key requirements in the ERA of GMOs [3]. In addition, it is recognised that biodiversity levels vary between agro-ecological regions [3]. Receiving environments are defined by the GM plant itself, i.e. the crop species plus the transgenic trait, the management systems within which the GMO operates and the geographical zone where the GMO is released [3]. Defining representative receiving environments does not only require the knowledge of the specific environmental and agronomic conditions of the specific region, but also implies the consideration of regional protection goals as well as differences in the baselines of species populations, ecological functions or other regional specificities.

#### Analysis of the guidance and suggestions for improvements

**Fig. 2:**
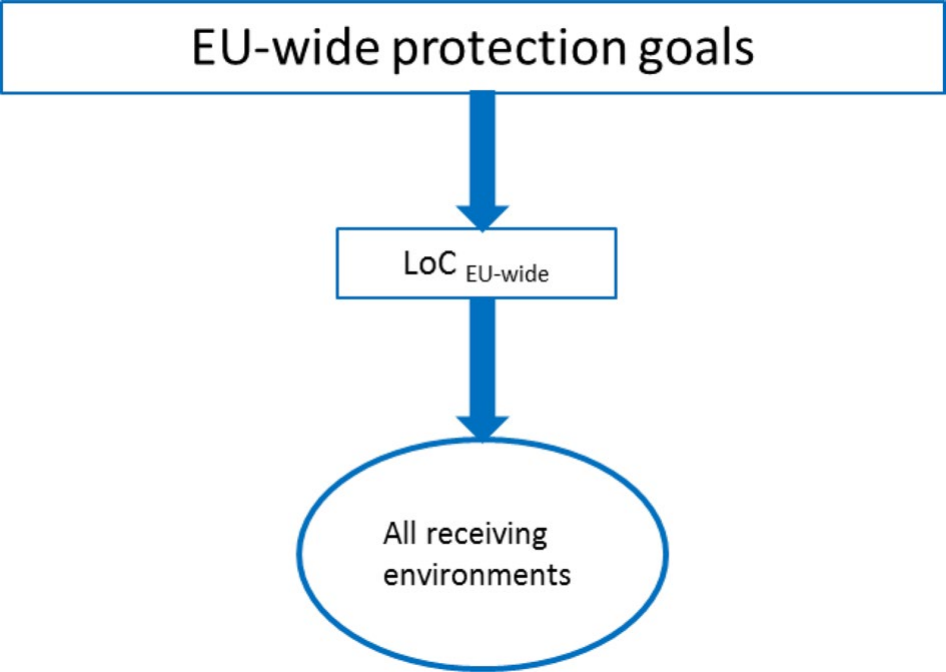
The LoC concept and the receiving environment (own interpretation from the EFSA guidance [3])

**Fig. 3:**
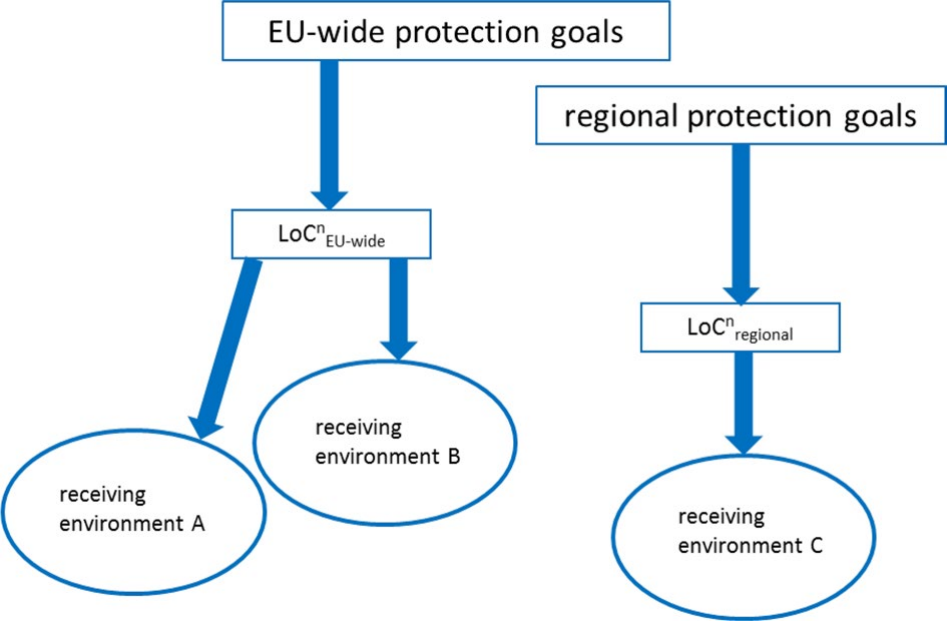
Possible regionalisation of the LoC concept. In this example, two sets of LoCs (LoC^n^) are defined for three receiving environments A, B and C. ${\text{LoC}}_{{_{\text{EU-wide}} }}^{\text{n}}$ are valid for the receiving environments A and B, whereas ${\text{LoC}}_{\text{regional}}^{\text{n}}$ are valid for receiving environment C only

According to the EFSA guidance, LoCs are directly derived from EU-wide protection goals and are valid for all receiving environments (Fig. 2). An important aspect so far left unconsidered by the EFSA guidance refers to the necessity to integrate regional specificities of the receiving environments into the LoC concept. A link between any damage thresholds defined at the EU level (i.e. the ${\text{LoC}}_{\text{EU-wide}}^{\text{n}}$) with the specificities of the receiving environments needs to be established (Fig. 3), thereby accounting for differences in environmental effects of the same GMO in different regions, e.g. due to large-scale land-use changes [20] or because of interactions with GM plants that are already cultivated in a specific region [3]. This may be achieved by modifying the ${\text{LoC}}_{\text{EU-wide}}^{\text{n}}$ according to individual conservation priorities of EU Member States resulting in a regionally adapted damage threshold (the LoC_regional_).

In this context, it has to be kept in mind that the definition of the receiving environment depends on the area of risk that is considered during the ERA. Receiving environments may be defined differently if potential effects on the crop management system are considered or if potential effects on non-target organisms are assessed. For example, considering changes in management practices for GM cotton in Europe may require defining different receiving environments in Greece and Spain, the two main cotton-producing countries in the EU: although Greece and Spain belong to the same biogeographical region, differences between these countries with regard to the occurrence of pathogenic fungi, seed treatments, soil preparation and weed management have been reported [21]. Therefore, regional differences in agricultural management measures such as irrigation intensity, crop rotation or weed and pest management practices have to be considered. In contrast, for the evaluation of non-target organisms, the receiving environments will be rather based on geographical zoning concepts [22] in order to account for differences in their occurrence and their regional relevance [23].

Alternatively, a regionalisation of damage thresholds may also be achieved after the authorisation of the GMO at Member State level, e.g. via the adoption of national opt-out measures restricting or prohibiting the cultivation of a GM crop according to Directive (EU) 2015/412.

### The role of the LoC in the stepwise testing approach of the ERA of GMOs

#### The EFSA guidance

An important feature of the ERA of GMOs is that hypotheses on adverse effects of the GMO on the environment are tested, progressing from worst-case scenarios under laboratory conditions to field trials under realistic environmental conditions [3]. In this context, EFSA refers to ‘tiered testing’, with tier 1a and 1b referring to in vitro or *in planta* laboratory tests, while tier 2 refers to semi-field tests (i.e. outdoor tests with some containment) and tier 3 to field tests [3]. In the scientific literature, there is a lot of controversy about how the tiered testing approach in the ERA of GMOs is to be applied and which kind of studies and data are necessary for each tier [6, 20, 24, 25].

**Fig. 4:**
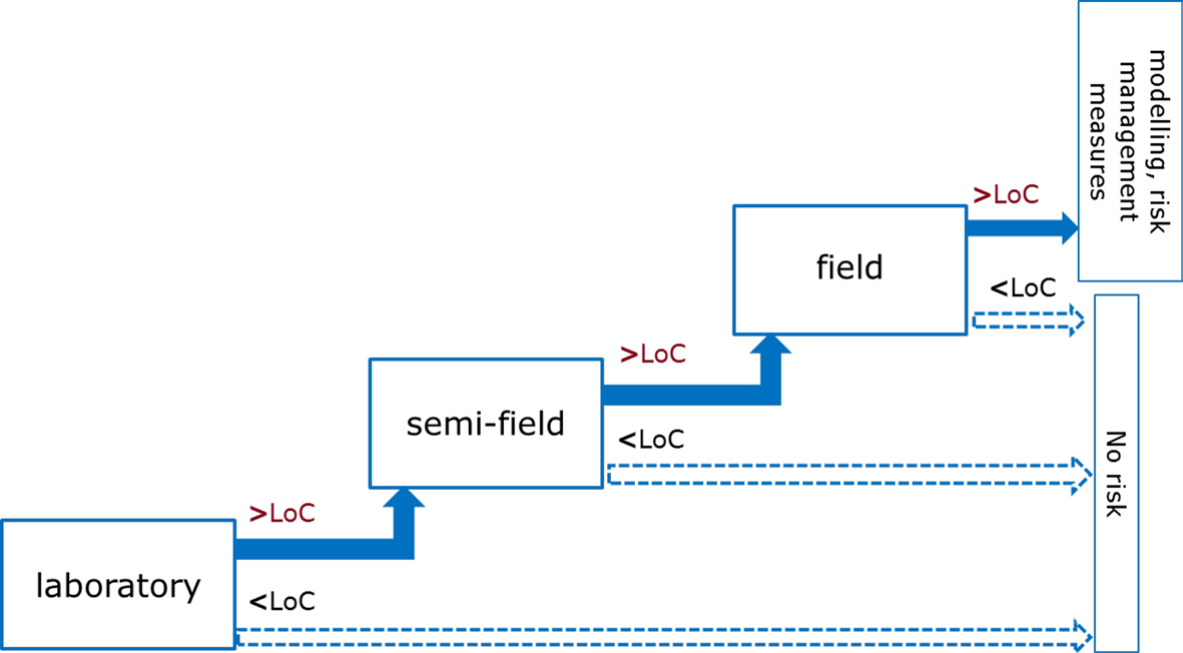
Limits of Concern and consequences for the stepwise testing strategy in the ERA of GMO as interpreted from the EFSA guidance [3]. *Filled arrows* indicate proceeding to the next (higher) tier, if the LoC is exceeded at the preceding tier. *Dashed arrows* indicate the conclusion of no risk and testing ends if the LoC is not exceeded

EFSA points out that LoCs should act as trigger values within the tiered testing approach. If an LoC is exceeded at a specific tier, then further tests at higher tiers or modelling and scaling up of effects are considered necessary [3]. Consequently, the LoC can be understood as a decision criterion indicating the necessity of further tests at higher tiers in case the LoC is exceeded. As long as EFSA does not explicitly state the consequences in case a LoC is not exceeded, the present provisions in the guidance can be interpreted in a way that the non-exceedance of a LoC is a stop criterion for the ERA, meaning that no further testing at higher tiers is considered necessary in order to conclude on the environmental risk of the GMO (Fig. 4).

#### Analysis of the guidance and suggestions for improvements

In the context of the tiered testing approach, the role of a LoC and the consequences of its exceedance are still to be clarified. Directive 2001/18/EC is the legal basis for the ERA of GMOs in the European Union and establishes the step-by-step principle. This principle allows for a gradual reduction of the containment of a GMO and the upscaling of its release only, if the evaluation of earlier steps indicates that the next step can be taken safely (Directive 2001/18/EC, Preamble 24 and 25). Although this principle is not a legal requirement for the authorisation, it is a general principle for the GMO risk assessment. It indicates that the reduction of the containment of a GMO should be accompanied by the build-up of knowledge about the performance and risks of that GMO [26]. In addition, it can be viewed as an additional tool to structure the collection of information for GMOs [26].

In accordance with the step-by-step principle of Directive 2001/18/EC, the role of the LoC in the ERA of GMOs should go beyond any type of stop criterion. As mentioned before, the idea and the purpose of the step-by-step principle are at least twofold: (i) to gradually decrease the containment of a GMO during testing and (ii) to simultaneously build up specific knowledge and reduce uncertainties about environmental risks related to the release of a GMO. These ideas would be compromised, if ERA testing was stopped in case the LoC was not exceeded already at a lower tier (e.g. a laboratory study), because then any uncertainties regarding environmental risks would be dismissed already at this early step of the ERA. This ‘*trade*-*off between simplicity of the ERA and uncertainty*’ [20] should therefore not be reinforced by a concept that allows for using LoCs as stop criteria.

It also has to be considered that LoCs for different studies with different containments of a GMO may not necessarily be related to each other as the studies themselves serve different purposes [3, 7]. LoCs for field studies will be related to the assessment of changes in abundance of individual species or species guilds, the evaluation of multi-trophic interactions, or ecosystem services (e.g. biological control). Therefore LoCs defined for field studies reflect more directly environmental damage thresholds for protection goals. In addition, LoCs for field studies should consider that effects observed in ecologically more realistic scenarios will have stronger consequences with respect to their relevance for the protection goals in question. However, effects occurring at larger scales (e.g. at landscape or regional level) or combinatorial effects (e.g. via the interaction of two or more environmental stressors such as *Bt* toxins) cannot be anticipated at field testing level [19]. For example, effects and processes at landscape-scale may have profound consequences on biodiversity [see e.g. 27] and such effects can only be accounted for by extrapolations using modelling or upscaling approaches in the ERA [see 19 and references therein]. Therefore, any LoCs for field level testing have to allow for potential large-scale implications of the effects observed at small-scale.

Lower tier tests such as laboratory tests serve to identify hazards (e.g. the sensitivity to a *Bt* toxin), clarify exposure routes or assess the extent of severity of an effect at single-species level under worst-case conditions of exposure and best-case environmental conditions, but often neglect ecological realism [20]. Therefore, LoCs for laboratory tests have a more indicative value as their role is to put the risk posed by a hazard into a broader context (e.g. the toxicity of a specific *Cry* toxin in comparison with other *Cry* toxins). There is insufficient knowledge in how far effects seen in ecotoxicological laboratory studies can predict the likelihood of adverse effects in field experiments [28–30]. In the ERA of plant protection products, trigger values for the two standard test species *Typhlodromus pyri* and *Aphidius* spp., tested with standard protocols in laboratory tests, have been validated by (semi-) field data [31]. For the ERA of GMOs, such a validation has not been made for any of the species tested in the laboratory. Also, standard test protocols and criteria are not yet commonly used, although recommended [32]. As long as certain minimum requirements for laboratory and field testing for GMOs are not met [6, 33], and validation of laboratory results in the field has not been done, LoCs should not be used as trigger values allowing risk conclusion from lower tier tests only.

**Fig. 5:**
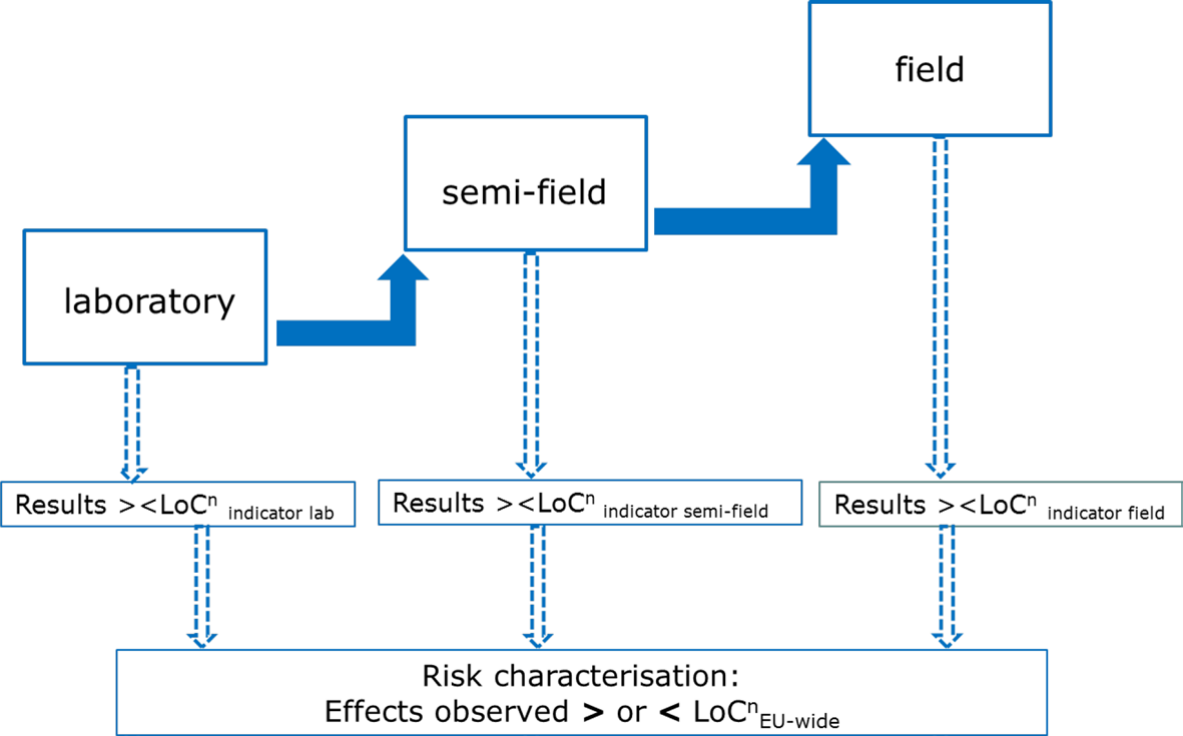
Limits of Concern and the consequence for the stepwise testing strategy in the ERA of GMO—own view. *Filled arrows* indicate the proceeding to the next (higher) tier, irrespective of whether or not the ${\text{LoC}}_{\text{indicator}}^{\text{n}}$ has been exceeded. *Dashed arrows* indicate that results from the testing are compared with the ${\text{LoC}}_{\text{indicator}}^{\text{n}}$ (exceedance/non-exceedance) at each tier and then integrated in the risk characterisation step of the ERA

Therefore, a more precautionary approach to the LoC concept would be to use the results (exceedances and non-exceedances of the LoCs) from all test tiers to inform the risk characterisation and to conclude whether the observed effects tested in the different tiers fall below or above the set of ${\text{LoC}}_{\text{EU-wide}}^{\text{n}}$ relevant for the respective protection goal (Fig. 5). Such a comprehensive approach would also correspond to EFSA’s description of the risk characterisation step in the ERA. In this step, which is carried out after ERA testing, an assessment should be made whether an observed effect falls within the LoC, thereby assessing its biological relevance [3, 7]. In this regard, it has to be kept in mind that usually a range of indicators are assessed at different tiers during the ERA, using several different measurement endpoints (lethal and sub-lethal parameters, population parameters, etc.) for different taxa and possibly their different development stages (e.g. when assessing effects on non-target butterflies). Comparing results from testing with the ${\text{LoC}}_{\text{indicator}}^{\text{n}}$ for each of the measurement endpoints will give a first indication of the risks to a specific indicator used for testing. However, results from all studies carried out at all steps (exceedance and non-exceedances of all ${\text{LoC}}_{\text{indicator}}^{\text{n}}$) will have to be integrated in the risk characterisation in order to conclude on the environmental risk for a particular indicator and, consequently, for the related protection goal.

### The link between the LoC concept and the comparative safety assessment

#### The EFSA guidance

The comparative safety assessment plays a major role in the whole risk assessment approach as it aims to identify differences between the GMO and its non-GM comparator due to either intended or unintended effects of the genetic modification [1, 3, 34]. Not only the differences between the GMO and the non-GM comparator need to be assessed, but also the so-called ‘equivalence limits’ need to be defined [5]. In general, when setting equivalence limits, the variability between commercial crop varieties is used [5]. In this context, reference to the LoC concept is implicitly made by stating that ‘*any significant differences or non*-*equivalences need to be followed up by an assessment of their biological or toxicological relevance, taking safety limits into account*’ [5]. EFSA thereby clearly recognises that the results of the comparative safety assessment are also relevant for the LoC concept, because statistically significant differences and non-equivalences may indicate unintended effects due to the genetic modification [3].

The ERA of GMO applications does not link the results of the comparative safety assessment, i.e. identified differences in parameters between the GMO and its non-GM comparator, with any ‘safety limits’. The results of the comparative safety assessment carried out for the food-feed risk assessment are usually presented in the ERA, but devoid of any evaluation whether they are of environmental relevance or not [35]. For example, detected statistically significant differences in anti-nutrient levels between GMOs and their non-GM comparators are not related to environmentally relevant levels of anti-nutrients affecting non-target organisms. Plant lectins constitute anti-nutrients in crops which are often assessed in the comparative safety assessments of GM soybean. Lectins are known to play an important role in the defensive and communication systems of plants [36, 37] and are known to affect arthropods [38–41].

#### Analysis of the guidance and suggestions for improvements

The comparative safety assessment is a specific concept introduced by EFSA in its guidance documents for the ERA of GMOs [1–3]. Its basic assumption is that conventional plants are familiar and therefore any novel plant variety (e.g. a GM plant) is compared to conventional plants for a range of molecular, agronomic and compositional assessments [35].

**Fig. 6:**
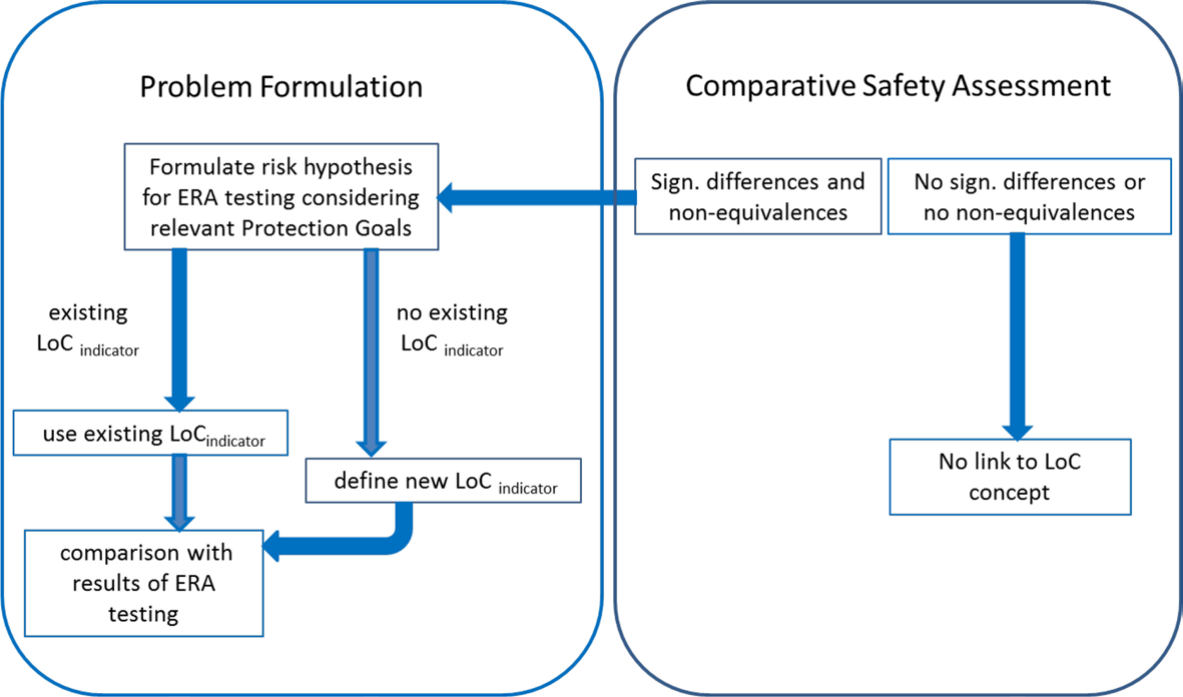
The relationship between the LoC approach and the comparative safety assessment in the ERA of GMPs. *Arrows* indicate consequences or necessary actions

The underlying assumption behind the concept of the comparative safety assessment is that traditionally cultivated plants are considered safe due to a history of safe use for human and animal consumption [2, 4]. This concept has been widened to include environmental effects as it is assumed that traditionally cultivated crops *‘…have gained familiarity for the environment*’ [5]. While the history of safe use is of relevance for traditional food and feed, a history of environmentally safe cropping cannot be established. Environmental impacts of conventional farming practices can be adverse and substantial, well documented e.g. by the decline in farmland bird populations in post-war Europe due to agricultural intensification [42, but see also 43, 44]. The introduction of provisions under the EU common agricultural policy, such as cross-compliance requirements, compensations for voluntary measures in the context of the rural development pillow as well as recently greening requirements, aims at the improvement of the environmental performance and increased sustainability of current agricultural practices in the EU. Consequently, a clear conceptual distinction is needed between the risk assessment for food and feed purposes with equivalence limits based on the natural variability of non-GM comparators, and the environmental risk assessment of GMOs using independent and protection goal-derived LoCs [45]. If the LoC concept is to be made operational, then a separate evaluation of the results of the comparative safety assessment for the ERA is needed, scrutinising (i) whether the assessed components cover sufficiently environmentally relevant plant compounds for the GMO in question and (ii) whether detected differences between GMOs and their non-GM comparators are of environmental relevance. Any detected significant differences or non-equivalences in the concentration of compositional parameters in relevant plant tissues need to be scrutinised whether they may affect relevant protection goals. This can be achieved by establishing a causal link between the environmentally relevant parameters and any existing LoCs for the protection goal in question. This approach is not only needed for compositional aspects of the GMO but also if differences in agronomic practices such as the application (e.g. numbers and frequency) of herbicides to be used with herbicide-tolerant crops are identified. A risk hypothesis has to be formulated to test whether the identified hazard falls below the existing LoCs for a relevant protection goal. Otherwise, it may be necessary to define a new LoC specifically adjusted to the hazards identified from the comparative assessment (Fig. 6). Therefore, the LoC concept has to be further specified and the ERA guidance has to be further developed in order to provide sufficient guidance for applicants and risk managers.

### LoCs for long-term effects

#### The EFSA guidance

Risks associated with the cultivation of GMOs may not only refer to direct and immediate effects of the GMO on the environment, but also to delayed or long-term effects, e.g. due to changes in the cultivation or management techniques of the GMO compared to its non-GM counterpart (Directive 2001/18/EC). The term ‘long-term effects’ refers to effects that occur up to 10 years after introduction of a new GMO (corresponding to the time of consent), but the time scale may be extended if perennial species are considered [3]. Long-term effects also cover effects that may occur through the persistence of GM progeny, e.g. as volunteers and ferals [3].

For changes in cultivation and management techniques due to the introduction of a GMO, EFSA suggests the use of a scenario analysis in order to assess the magnitude of effects in the ERA of GMOs, and at the same time recognises the difficulty in predicting such effects [3]. Furthermore, modelling and desk-based studies including information of existing examples from other regions with GMO cultivation are considered necessary for the assessment of such long-term risks [3].

#### Analysis of the guidance and suggestions for improvements

EFSA does not specifically address the use of LoC when long-term effects are assessed. However, as the setting of LoCs is an inherent requirement of the problem formulation of the ERA, they are also of relevance for long-term effects. LoCs have to be defined for long-term effects if they are likely to occur and if appropriate risk hypotheses can be formulated. Long-term effects often occur under novel agro-ecological conditions, which were not encountered during the ERA or which are difficult to be tested in the ERA. An example is the resistance development of weed species to non-selective herbicides applied with the corresponding herbicide-tolerant GMO. Resistance development does not occur immediately when herbicide-tolerant GMOs are cultivated, but after several years of extended use of the complementary herbicide [46]. Resistance development of target species is clearly recognised as an adverse effect in the ERA of insect-resistant GM maize requiring risk mitigation measures as well as case-specific monitoring [33]. For insect-resistant GM maize, the aim is to prevent the occurrence of resistance in the respective target organisms, thereby defining a damage threshold. In contrast, no such thresholds for the resistance development of weeds due to the application of the non-selective herbicide in herbicide-tolerant crops have been defined yet [33].

In the ERA practice, it will not be possible to assess the exact type and extent of the adverse effects that the novel management and cultivation practices associated with herbicide-tolerant GMO cultivation may have. Nevertheless, the formulation of LoCs will be needed also for these types of effects [see also 18]. Risk management measures and a well-designed post-market environmental monitoring are needed in order to evaluate whether any LoC for long-term effects are exceeded during cultivation. The efficacy of specific risk management measures (e.g. weed resistance management measures) needs to be evaluated at least during the post-market monitoring period, usually 10 years after consent, and ideally longer. Any adverse effect observed during the post-market monitoring period (e.g. the occurrence of resistant weed species) needs to be scrutinised whether it has been caused by the cultivation of the respective GMO and whether it falls within the defined LoCs.

### Discrimination of LoCs for non-target organisms

#### The EFSA guidance

When carrying out the ERA of GMOs, the obligation to suggest specific LoC values lies with the applicant. He is requested to determine the level of environmental protection by defining a LoC for each measurement endpoint tested [3]. However, EFSA indicates specific LoC values to be used as a starting point. For laboratory studies, EFSA suggests an effect size of 20%, while for semi-field testing 30% and for field studies 50% effect sizes are proposed as possible LoCs [3, 7].

#### Analysis of the guidance and suggestions for improvements

The scientific rationale behind the values suggested by EFSA remains largely unclear. EFSA points out that an effect size of 20% is ‘*often taken as the trigger value for further, higher*-*tier studies*’ [3]. No further indication is made in which context these trigger values are to be used.

According to the currently used guidance document for plant protection products, the trigger value used for first-tier testing of non-target arthropods is a hazard quotient (HQ), which is defined as the specific application rate of the product divided by the application rate causing 50% mortality in the laboratory [47]. If for a certain plant protection product, the HQ value is equal to or exceeds the trigger value for one or both of the two indicator species, a potential hazard to non-target arthropods is concluded, triggering further data requirements or risk management measures [48, 49]. The trigger values have been developed and are recommended for the two indicator species *Aphidius* spp. and *Typhlodromus pyri*, after they were validated by (semi-)field data and for a wide range of plant protection products [31, 47].

However, for GMOs, no such validation studies have been conducted so far (see also “The role of the LoC in the stepwise testing approach of the ERA of GMOs” section). It is questionable whether effect sizes of 20% or smaller are reliable predictors of harmless effects on a range of non-target organisms in the field, when testing the GMO at lower tier, e.g. in the laboratory. In particular, if the 20% value is used for laboratory mortality data, it would also have to cover sub-lethal and reproductive effects that might occur under field conditions. Additionally, any decision on acceptable effect sizes for laboratory testing should take into consideration that, e.g. for mites, mortalities of 20–25% in the control group are accepted validity criteria for ecotoxicological effect testing of chemicals or plant protection products [50, 51]. Although LoCs should be set independently from acceptable control mortalities for standardised ecotoxicological testing protocols, these must be kept in mind when deciding on acceptable effect sizes for laboratory tests using non-target organisms.

EFSA also suggests using a 50% effect size as a possible LoC value for field studies, however without specifying the temporal and spatial validity [3, 7]. EFSA refers to a study conducted in the context of the British Farm Scale Evaluations in which weed density, biomass and species diversity between herbicide-tolerant GMOs and fields with conventional management in a split-field design was tested [52]. The design of the study allowed to detect multiplicative differences of *R* = 1.5-fold, indicating a 50% increase or a 33.3% decrease [53]. In the ERA of plant protection products, an arbitrary 50% effect threshold value is used for the evaluation of effects on non-target arthropods in semi-field studies, mostly due to statistical reasons [54]. The proposed 50% effect size as a field LoC seems to comply with statistical constraints rather than with biological needs of the affected ecological entities. Considering acceptable effect sizes of 50% for cultivated GMP would counteract current efforts to halt the loss of farmland biodiversity. Acceptable effect thresholds have to take into account that even current agricultural practices are among the most important pressures on terrestrial biodiversity and ecosystems [43, 44]. Considering that the type of agriculture used as comparator for the evaluation of adverse impacts of GMOs is causing farmland biodiversity loss already, additional impacts due to the proposed 50% effect size for GMO cultivation cannot be considered acceptable.

In the ERA of plant protection products, decisions on the acceptability of adverse effects observed in the field are made case-by-case, also accounting for differences of effects on different arthropod taxa, considering the mobility of the species, its reproduction time and affected development stage [47, 51, 54]. When defining a LoC_indicator_ for GMO field studies, acceptable adverse effects have to be discriminated depending on which taxon or functional group of non-target organism is considered.

In addition, population parameters and the potential for recovery are important aspects [7]. For non-target organisms, EFSA specifically refers to a linkage between significant effects at the population level of a non-target organism and the defined LoC [7]. When estimating the natural variability of populations of non-target organisms, it is crucial to consider population trends over extended time periods as they may give a different picture than annual fluctuations. For example, annual declines of farmland birds in the United Kingdom range from 2 to 10% but 10-year population declines of many species exceeded 10% with some species experiencing declines of up to 40% [55]. Therefore, defining the significance of an observed adverse effect is not feasible without setting the spatial and temporal context. This may be hampered by a lack of knowledge on the biology of the species or reasons for its decline. When defining a LoC, it is not only important to discriminate between different taxa but also to define the temporal and spatial boundaries for which a LoC is supposed to be valid. In addition, if population parameters are shifting with time, then general downward trends may not be taken into account. Consideration of shifting baselines has been identified as an important factor for setting of reference points and targets for rehabilitation measures in fisheries science [56]. Similarly, declines in the population trends of major farmland taxa have been described since the beginning of the last century [57]. Such shifts in population trends need to be accounted for if acceptable thresholds for environmental harm are to be defined. This can be done by either regularly amending the LoC according to the knowledge about the population status (relative LoC) or fixed LoC values are set, defining the minimum environmental quality to be preserved, independent of the general population trends observed (absolute LoC).

### LoCs for species of conservation concern

#### The EFSA guidance

Many of the protection goals which need to be considered during the ERA of GMOs relate to species protected at EU level, e.g. by the FFH Directive and Birds Directive [3]. Also when categorising and ranking non-target focal species for ERA testing purposes, endangered or threatened species need to be addressed [3, 7].

In 2015, EFSA explicitly addressed endangered species and their role in the ERA of GMOs, plant protection products and feed additives in two guidance documents, [13, 58]. With respect to the magnitude of the acceptable effect observed in the ERA, EFSA considers the level of endangerment as a critical factor, also recognising that this can vary regionally [13]. In addition, the importance of critical sub-populations of endangered species is also addressed [13].

#### Analysis of the guidance and suggestions for improvements

When defining LoCs, it is necessary to differentiate between species of conservation concern and those species that are considered ‘common’ in the agro-environment. Common species are considered to be widely distributed, to occupy different habitats and to have large population sizes [58]. Species of conservation concern may be either protected by EU law, nationally or regionally. However, species occurring in and adjacent to agro-ecosystems may be rare, endangered or threatened without being protected by a regulatory act [59, 60]. Many of these species are covered by Red Lists, either regionally, nationally, at EU level [61] or even globally [62]. Consequently, the level of protection or endangerment may not be uniform across the EU and individual Member States may not accept an EU-wide threshold for environmental harm (the LoC_EU-wide_) for a particular species of conservation concern.

Non-target butterflies are often highlighted as prominent examples of protection goals for the ERA of GMOs expressing lepidopteran-specific toxins (e.g. *Bt* crops). Lepidopteran non-target species may ingest the *Bt* toxins expressed in parts of the GM plants and be subject to sub-lethal or lethal effects [63]. About one-third of European butterflies show declining populations with agricultural intensification as the major cause for habitat loss or degradation [61].

In this context, it has been questioned whether species of conservation concern are more vulnerable to potential stressors such as GMOs than other species [58]. The concept of ecological vulnerability comprises the factor sensitivity and exposure to a stressor as well as the potential for recovery [58]. When considering non-target butterflies of conservation concern, it can be concluded that the knowledge on their vulnerability to *Bt* crops as a potential novel stressor in the agro-environment is largely limited. First, knowledge on the sensitivity of different lepidopteran species to *Bt* toxins is still limited [64, 65] and evaluations of potential effects of *Bt* toxins on different species are derived from modelling using data from a few surrogate species [66]. Second, knowledge on the actual exposure of lepidopteran species to *Bt* toxins under field conditions and methods for modelling exposure for ERA purposes are a constant matter of debate in the scientific literature [65, 67–69]. With respect to the third aspect of ecological vulnerability, parameters to assess recovery of butterflies from adverse effects due to *Bt* toxin ingestion have so far not been included in laboratory or field evaluations [63]. From a precautionary view, population recovery should not be considered in the ERA for species whose population status is already outside of the ‘normal operating range’ [58]. In accordance with current ERA recommendations for plant protection products, the concept of recovery is applicable for in-crop assessments but not for the off-crop area where no or only transient effects are acceptable for non-target arthropods [70]. The off-crop area is particularly relevant for many lepidopteran species of conservation concern that occupy natural habitats interlinked into the agro-environment where *Bt* crops may be cultivated [71–74].

If certain lepidopteran non-target species have been attributed a higher value than other species occurring in agricultural landscapes, then any further pressures put on their populations which may lead to the deterioration of their populations or, in the worst case, to their extinction must be avoided. The LoC concept must therefore provide a possibility for setting more conservative LoCs than for ‘common’ species [16], e.g. by accepting no or only negligible effect sizes for species of conservation concern due to the cultivation of GMOs.

## Conclusions and outlook

Specifying what constitutes environmental harm is a highly political issue and depends not only on European policies but also on national priorities in conservation efforts. The preceding years have shown that carrying out the ERA of GMOs without a definition of biologically relevant effects and of environmental harm led to substantial controversies about their environmental safety. It also led to the invocation of national safeguard clause measures or emergency measures under Article 23 of Directive 2001/18/EC and Article 34 of Regulation (EC) 1829/2003, respectively. Some of these measures were argued with the potential adverse effects of insect-resistant maize on non-target species [e.g. 75]. The underlying controversy often derived from a different perception of what constitutes environmental harm for these species [76]. While science can support decisions on the relevance of adverse effects observed, it cannot make any of the normative decisions on what, where and when to protect. Even though definitions of environmental harm to protection goals are mostly lacking, thresholds for environmental harm for ERA purposes need to be defined. Similarly, the lack of scientific knowledge regarding what constitutes environmental harm should not prevent the formulation of Limits of Concern. The definition of LoCs should not only take into account the scientific uncertainties, but has to consider that current pressure due to agricultural practices on European farmland biodiversity is already extensive, thereby calling for LoCs for GMOs that do not go beyond current effect levels due to conventional agriculture. In any case, the political and scientific justifications behind the decisions when defining Limits of Concern must be made transparent.

In the future, the LoC concept will be useful as it opens up the ERA for the integration of protection goals and puts pressure on EU Member States to specify their environmental policy objectives. In order to be a workable approach, thresholds for environmental harm to be used for ERA purposes need to be spatially and temporally differentiated from thresholds of harm at the protection goal level. In this context, it is important and needs further attention to recognise that there are various receiving environments across Europe which have their own specificities. If biodiversity levels can be clearly distinguished and protection goal priorities differ between these receiving environments, then this must be accounted for when defining what adverse environmental effects are acceptable, e.g. by the use of regionally adapted LoCs. This also implies that LoCs have to discriminate between species and ecological functions rather than using standardised threshold values across taxa and functions. In this context, species of conservation concern need a separate consideration acknowledging their outstanding role in the conservation of European biodiversity.

For the operationalisation of the LoC concept, it has to be recognised that many profound ecological questions still remain unanswered, e.g. what level of biodiversity is needed to sustain certain ecosystem functions or what the consequences of certain adverse effect sizes of environmental stressors on the biodiversity in arable ecosystems are. There are still open questions on how to predict scale-dependent impacts of GMP cultivation across landscapes or regions during the full authorisation period of GMPs, which currently can only be addressed via modelling. Any decisions on LoCs for the ERA must therefore be aware of the potential consequences of acceptable effect sizes for the relevant ecological entities and ecosystem functions and services if large-scale and long-term GMO cultivation is envisaged. Consequently, the responsibility to decide on LoCs cannot be restricted to applicants alone, but must be divided between different stakeholders involved, such as risk managers, risk assessors, applicants and the scientific community. This will also serve the aim to apply comparable LoCs for the ERA of GMOs with similar traits.

In order to arrive at a common understanding among applicants, risk assessors and risk managers, the question of what the consequences are if LoCs are exceeded has to be answered before the LoC concept is to be made operational, Tentative interpretations bear the risk of misusing the LoC as a stop criterion for the stepwise testing approach of the ERA thus again fuelling controversies about fundamental risk assessment approaches of GMOs. Instead, the opportunity should be taken to strengthen the stepwise ERA approach and thus increase confidence in risk conclusions.

Further clarifications are needed if results of the comparative safety assessments are to be used for the LoC concept. These include the establishment of links between environmentally relevant parameters assessed in comparative safety assessment and any LoCs defined. Only by using LoCs as suitable reference points, the observed differences between the GMO and its conventional counterpart can be put into context in terms of environmental safety. Here a strict differentiation between the food-feed risk assessment and the ERA has to be made.

LoCs will also be relevant for the question of how to deal with long-term effects of GMOs which cannot be tested in the ERA. Any LoCs defined for such effects need to be combined with corresponding risk management measures while simultaneously strengthening post-market monitoring.

The setting of LoCs will not be an uncontroversial issue. However, defining LoCs opens the way for discussions on what to protect, where and when, in European agro-ecosystems. First steps have to be made and specific LoCs have to be suggested in order to start discussions based on which risk managers will have to rethink their priorities, not only with respect to conservation goals, but also regarding trade-offs between different ecosystem services provided by the agro-ecosystem.

## Methods

In 2013, the German Federal Agency for Nature Conservation commissioned the project ‘Limits of Concern for the Risk Assessment of GM plants’. The aim of this project is the critical evaluation of the concept of Limits of Concern introduced by EFSA for the practical implementation in GMO risk assessment. The project is comprised of several work packages, amongst others the evaluation of environmental harm thresholds applied for regulated products other than GMOs, the realisation of stakeholder interviews and expert workshops for feedback and critical discussions on the concept. Within this project, selected GMO applications according to Regulation (EC) No. 1829/2003 were scrutinised with the focus on the consideration of the LoC concept. This could be expected because all applications were submitted in 2010 and later and therefore should follow EFSA’s revised ERA guidance. Since no applications for cultivation were available from this period, the focus was put on applications for import and processing only. The GMO dossiers were examined for the application of the LoC concept for environmentally relevant compounds assessed in the comparative safety assessment. Specifically, the application of GM soybean DAS44406-6 was selected due to the fact that the lectin content in this event, as compared to the non-GM soybean, was significantly different and non-equivalent (notification EFSA-GMO-NL-2012-106, technical dossier, pp 130).

In this article, we summarise results from these first work packages and scrutinise the LoC concept as outlined by EFSA [3, 7] by addressing fundamental questions of the concept in the context of GMO risk assessment. The focus is put on LoCs for GMOs intended for cultivation in the EU. In addition to these questions, open issues are identified which need to be solved in order to encourage the scientific discussion and make further progress in the practical implementation of the LoC concept in the ERA of GMOs. Results on the possibility to set LoCs for specific areas of risk will be published separately. During the course of the project and the expert workshops and interviews, the following key questions were identified and are addressed in this article:

- How can LoCs be derived from protection goals?
- Which role do LoCs play in the stepwise testing approach of the ERA of GMOs?
- What is the relationship between the comparative safety assessment and LoCs?
- Should LoCs be set for long-term effects?
- Are the LoC values for non-target organisms suggested by [3] practicable and reasonable?
- Should there be different LoCs for species of conservation concern and non-protected species?

These key questions are discussed in various chapters of the “Results and discussion” section. First, the important aspects of LoC as outlined by the relevant EFSA guidance documents are presented in the initial sub-chapters “The EFSA guidance” of each of the seven chapters. In a second step, these aspects are critically scrutinised and suggestions for improvements are made which are considered crucial for the operationalisation of the concept (sub-chapters “Analysis of the guidance and suggestions for improvements”).

Different acronyms for Limit of Concern will be used throughout the text. LoC refers to the general term as described in the ERA guidance documents [3, 7]. LoC^n^ refers to a specific set of several Limits of Concern. LoC_EU-wide_ specifies Limits of Concern that refer specifically to environmental protection goals at EU level. LoC_indicator_ delineates Limits of Concern at the level of ERA testing. Further explanations are given in the text.

## Abbreviations

EFSA: European Food Safety Authority
ERA: Environmental Risk Assessment
GMO: genetically modified organism
GM: genetically modified
LoC: Limit of Concern
